# Isolation and characterization of thermotolerant hydrocarbon degrading bacteria which sustained the activity at extreme salinity and high osmotic conditions

**DOI:** 10.22099/mbrc.2024.49747.1946

**Published:** 2025

**Authors:** Saman Hosseini, Rouhallah Sharifi, Alireza Habibi, Ali Beheshti-AleAgha

**Affiliations:** 1 Department of Plant Protection, College of Agriculture, Razi University, Kermanshah, Iran; 2 Faculty of Petroleum and Chemical Engineering, Razi University, Kermanshah, Iran; 3 Department of Soil Science, College of Agriculture, Razi University, Kermanshah, Iran

**Keywords:** Bioremediation, Indigenous bacteria, Abiotic stress, Extreme environment, PCR

## Abstract

The bioremediation method is considered an economical and environmentally friendly strategy for the remediation of oil-contaminated soils. However, some oil field areas have extreme environmental conditions that make it difficult to establish microbes for bioreme-diation. In this study, bacteria were isolated from oil-contaminated soils of the Dehloran oil fields, which have very harsh soil and weather conditions. Soil samples were collected from two highly contaminated mud pits. The petroleum content and physicochemical characteristics of the soil were investigated. Soil samples pollution were about 8%, sandy and alkaline, and their EC reached up to 125.6 ds/m in some samples. The isolated bacteria were screened according to their ability to grow on the M9 mineral medium containing crude oil as the sole carbon source. Moreover, their physiological characteristics in diesel degradation were investigated. The phenotypic, biochemical, and molecular characteristics of selected isolates and their stability under extreme conditions such as drought, salinity and high temperatures were investigated. Two isolates NC39 and NB391 showed the highest ability in diesel degradation. The results of 16SrRNA sequencing showed that NC39 isolate had 98% similarity to *Pseudomonas* sp*.* and isolate NB391 belonged to *Pantoea* *agglomerans* with 99% similarity*.* These two isolates showed a high ability to tolerate high salinity (10%), temperature (50°C), and drought (-0.73 MPa) stress. Exploiting these extremophile strains is a promising tool in the bioremediation of oil-contaminated soils in extreme environments*.*

## INTRODUCTION

Environmental pollution is one of the most important, highly growing challenges in the modern world [1]. Petroleum pollutants and their derivatives all have destructive effects on human, plant, and animal health and cause disturbance in the ecosystem cycle [2, 3]. In addition to soil pollution, they cause atmospheric pollution by evaporation, and then by entering surface and underground water, they increase the dangers [4]. To protect the environment and human health, these dangerous pollutions must be removed, and methods such as physical, mechanical and chemical methods are used to remove hydrocarbon pollution from the soil. However, these methods are suitable only for high pollution levels, and they are not recommended for normal contamination because of their cost and disturbing effects on soil structure and the ecosystem [5]. Nowadays, the bioremediation method using bacteria has been used as an economic strategy for the remediation of soil contaminated with petroleum. This method is compatible with the ecosystem, speeding up the degrading process, cost-effectiveness, and degrading hydrocarbon pollutants into non-toxic substances [6]. 

Soil microbial diversity is influenced by environmental factors such as the availability of food resources in the short and long term. Opportunistic bacterial populations, with their remarkable and diverse abilities use different substrates for energy absorption and growth. Thus, adding petroleum hydrocarbons to the soil provides a new and abundant source of carbon to the environment, and bacteria with the ability to degrade it gain a new ecological advantage over other microbes in that environment [7]. Due to this compatibility, it can be seen that indigenous microorganisms have a greater ability to degrade petroleum hydrocarbons [8]. 

Various environmental factors can affect the degradation ability of bacteria, some of these factors can be controlled or modified, but others are difficult. The most important factors are soil texture and structure, pH, temperature, humidity, nutrient sources, salinity, and oxygen [9]. Biodegradation of petroleum hydrocarbons occurs in aerobic and anaerobic conditions, but aerobic biodegradation is usually faster than anaerobic biodegradation [9]. Temperature affects the metabolism of bacteria, their growth rate, the physicochemical state of pollutants, and the solubility of petroleum hydrocarbons, so bioremediation usually occurs more effectively under optimal temperature conditions (20-40°C) [10]. Water is essential for all life, so it is not surprising that very little bioremediation occurs in dry regions and polluted soils except during the rainy season [11]. However, even in temperate soils, the amount of water can significantly affect the structure and abundance of the soil microbial community [12]. Extreme changes in salinity are also other factors that can affect the ability of bacteria to bioremediation in such a way that salty environments reduce the solubility of hydrocarbons and oxygen in water, which has a negative effect on the ability of bacteria in bioremediation and perform essential metabolic functions, but bacteria adapted to saline environments (2.5 to 10%) are less affected [13, 14]. 

The present study was conducted to isolate and determine the efficiency of oil-degrading bacteria in an area with unfavorable soil and weather conditions in the west of Iran. In the given area, extensive soil contamination with a high concentration of petroleum compounds (up to 10%) has occurred. In addition, the air temperature in the region was high, rain precipitation was low, and the soil was very salty; these features are more or less present in most oil fields in Iran and the Middle East. This study aimed to isolate, identify, and screen bacterial isolates that degrade petroleum hydrocarbons, and further the resistance of the isolates to drought, salinity, and temperature stresses were investigated to obtain efficient and resistant degrader isolates against extreme environmental conditions.

## MATERIALS AND METHODS

**Soil sampling: **To isolate bacteria from the oil-contaminated soils of the Dehloran region, soil samples were collected and placed into sterile plastic bags and transferred to the laboratory. Additionally, the physicochemical characteristics of soil samples, including pH, soil salinity (EC), percentage of organic matter (OC), and soil type were investigated [15]. The sampling area had a hot and dry climate with an average annual rainfall of 158 mm.

**Isolation and purification of isolates: **To isolate the bacterial isolates from the soil sample, one gram of the soil sample was mixed well in 9 ml of sterile distilled water. Then, the dilution series was continued until dilution 10^-4^ due to the high salinity of the soil sample and the possibility of a low population of bacteria. Afterward, 0.1 mL from a suspension of each dilution was spread on the surface of a nutrient agar (NA) medium in three replicates, and the Petri dishes were incubated for 72 hours at 28°C. Isolates with different colony shapes were selected and purified in a NA medium. To preserve the isolates for a long time, the suspension of bacteria was prepared in nutrient broth containing 50% glycerol and stored at -80°C [16]. 

**Screening in petroleum solid medium: **To screen isolates that degrade petroleum hydrocarbons, M9 solid medium (NaCl, 0.5 g/L; Urea, 1 g/L; KH_2_PO_4_, 3 g/L; Na_2_HPO_4_, 6.78 g/L; MgSO_4_.7H_2_O, 0.101 g/L; Agar, 15 g/L) was prepared and after autoclaving at 121°C for 20 minutes, 1.5 g/L of sterilized crude oil (the only source of carbon) with a PTFE membrane filter with pore sizes of 0.22 µm, was added to the medium. The purified colonies were cultured on the M9 plate and incubated at 28ºC for 3-5 days [17]. 

**Drought tolerance test:** The ability of isolates to grow in different drought potentials was investigated in a solid medium [18]. First, the osmotic potentials of -0.05, -0.15, -0.3, -0.49, and -0.73 MPa were prepared with the addition of 56, 108, 163, 211, and 261 grams of polyethylene glycol (PEG 6000) in 1 liter of nutrient broth medium, respectively after autoclaving the nutrient broth medium according to the following formula:

*π* (bar) = -1.18 ×10^-2^ × *C* - 1.18 ×10^-4^ × *C*^2^ + 2.67 ×10^-4^ × *CT* + 8.39 × l0^-7^ × *C*^2^*T*

In this formula, *T *is temperature, which was considered to be 30ºC, *C* is the concentration of polyethylene glycol (PEG 6000)(g/L), and *π* is osmotic potential in terms of bar, which was converted into Mega Pascal after calculation [19]. Afterward, approximately 15 mL of a nutrient solution containing different concentrations of polyethylene glycol (PEG 6000) was spread on the surface of Petri dishes containing nutrient agar medium that was prepared in advance and had the same nutrient as polyethylene glycol nutrient solution. It was incubated at room temperature for 24 hours. It was kept until polyethylene glycol leaked into the solid medium, and the desired osmotic potential was created. After 24 hours, the colonies were cultured and incubated at 30°C. Results were recorded as growth or no growth [18]. 

**Salt tolerance test: **Salt tolerance of the isolates was assayed due to the high salinity of the soil in the sampling region. Nutrient agar medium was prepared with an electrical conductivity of 20, 60, 140, and 200 decisiemens per meter (dS/m) containing 1%, 3%, 7%, and 10% of NaCl, respectively, with the control treatment (without adding NaCl). After the culture of the colonies on the surface of the medium, the Petri dishes were incubated at 28°C for 72 h. The results were recorded as growth or non-growth in different salt concentrations [20]. 

**Temperature tolerance test:** To investigate the temperature tolerance of the isolates, the colonies were cultured on the nutrient agar medium and incubated at different temperatures of 35, 40, 45, and 50°C. The results were recorded as the growth or non-growth of isolates [16]. 

**Investigation of growth in the diesel-containing medium: **The efficiency of the selected isolates was investigated using diesel hydrocarbons as the sole carbon source. The experiments were carried out in 250-mL-autoclaved Erlenmeyer flasks containing 100 mL of M9 liquid medium and 5 g/L of diesel. Then, the Erlenmeyer flasks were inoculated with 1 mL (1% v/v) of suspension 10^7^-10^8^ CFU/mL of selected isolates. The flasks were incubated at 28ºC on the incubator shaker at 130 rpm for 7 days [17]. After 7 days, the medium was centrifuged at 6000 rpm for 10 min, the supernatant was discarded, and physiological serum was added to the pellet and mixed well. Then, the concentration of the suspension was measured at 600 nm. 

**Biochemical and molecular identifications: **Phenotypic and biochemical tests were performed to initially identify the isolates including; Gram reaction, colony shape, catalase, oxidase, urease, and oxidation and fermentation (O/F) [16]. 

According to diesel degradation, two efficient isolates were selected for molecular identification based on the 16SrRNA gene. For total DNA extraction, a suspension was prepared from the fresh culture of each isolate in 1.5 mL microtubes containing sterile distilled water. Then, the suspension was centrifuged at 8000 rcf for 2 min. The supernatant was discarded, and the bacterial pellet was resuspended in sterile distilled water. This step was repeated. A 200 µL of 0.1 M potassium hydroxide (KOH) solution was added to the pellet, and the microtubes were placed in boiling water at 95°C for 10 min. After becoming clear, the suspension was centrifuged for 5 min at 12,000 rcf, and the supernatant containing DNA was transferred to a new sterile microtube and stored at -20°C [21]. The 16SrRNA gene was amplified by polymerase chain reaction (PCR) using two universal primers 27F (5’-AGAGTTTGATCMTGGCTCAG-3’) and 1492R (5’-TACGGYTACCTTGTTACGACTT-3’) [22]. The final volume of the PCR reaction mixture was 25 µL, including 12.5 µL of master mix (2x) (Amplicon), 1.5 µL of each reverse and forward primer, 2 µL of sample DNA, and 7.5 µL of sterile distilled water. PCR cycles include: initial denaturation at 95°C for 5 min and then 30 cycles including denaturation at 94°C for 45 seconds, primer annealing at 55°C for 45 seconds, and extension at 72°C for 2 min, and the final extension was at 72°C for 10 min. The polymerase chain reaction was performed in a Biometra thermocycler (T-Personal, Analytik Jena, Germany). A 3 µL of PCR products were loaded on 1% agarose gel containing DNA Green viewer dye (Parstos Company) and electrophoresed for 45 min at 80 V. The PCR product was sent to Microsynth Switzerland for sequencing. 

The received sequences were manually edited using the Geneious Prime software (v2. 2019) and aligned with the reference sequences in the GenBank using the Basic Local Alignment Search Tool (BLAST). The sequences from this study and the reference sequences obtained from GenBank were first aligned using the MUSCLE method for phylogenetic analysis. The phylogenetic analysis was performed using the MEGA X software based on the neighbor-joining method (1000 bootstrap replicates).

## RESULTS

The physiochemical characteristics of soil samples, including pH, soil salinity (EC), percentage of organic matter (OC), clay, silt, and sand content, and as well as the soil texture types, are described in Table 1. Among the samples, the sample of A35 was taken from the non-contaminated area around the contaminated mud pits, and the sample of B38 was taken from the depths of the mud pit of well No. 38, where the contamination had not penetrated that far. These two samples represented the natural conditions of the soil in the region. These soils have a silty loam texture, and their pH was alkaline in the range of 8.42-8.48, and EC varied in ranging of 1.35-3.29 dS/m (Table 1). Organic carbon was very low, less than 0.5% of the soil. 

**Table 1:** Characteristics of oil-contaminated soil samples and the number of bacterial isolates obtained from the Dehloran region, Iran.

**Soil type**	**Sand **(**%**)	**Silt** (**%**)	**Clay **(**%**)	**EC** **(dS/m)**	**pH**	**OC **(**%**)	**Number** **isolates**	**Code of sample**
Sandy loam	72	18	10	1.35	8.47	0.49	8	A35
Sand	92	2	6	94.4	7.45	7.61	2	A39
Silty loam	32	58	10	70.9	7.46	2.05	1	A38
Sandy loam	82	10	8	3.29	8.42	0.45	8	B38
Loam	42	42	16	103.5	7.51	1.72	2	B39
Sand	96	2	2	47.4	7.81	8.19	5	C39
Sandy loam	70	20	10	125.6	6.67	7.8	1	D39
Loam	48	44	8	9.95	7.98	2.93	2	C38
Sandy loam	68	26	6	8.21	7.54	7.61	8	D38

The microbial population of these soils was low; however, it was more than the contaminated soils. Other soil samples were taken from the contaminated mud pits, which had different percentages of petroleum hydrocarbon and high salinity. Overall, the mud pit well No. 39 samples had more salinity and hydrocarbon pollution. Samples of B39 and D39 had very high salinity (103.5-125.6 dS/m), which can restrict biodegradation. Salinity was also high in other contaminated soil samples. The soil samples of A39 and C39 had a sandy texture with 92% and 96% of sand, respectively. Due to high amounts of petroleum hydrocarbons, the percentage of organic carbon in soil samples was high and reached up to 8.12%. In the contaminated soils, the pH was lower than in non-contaminated samples (Table 1).

From the contaminated soil samples, 37 bacterial isolates with different morphological characteristics were isolated. The number of isolates obtained from each soil sample is presented in Table 1. Among these isolates, 15 isolates showed the ability to grow in a solid oil medium containing 1.5 g/L of crude oil, which was used for further studies (a list of the selected isolates is given in Table 2). The performance of the 15 selected isolates to tolerate drought, salinity, and temperature stress are presented in Table 2. All isolates could tolerate drought up to the potential of -0.49 MPa; among them, only three isolates of NB83, NB833, and NB835 could not grow at -0.73 MPa. The results indicate the high capability of the isolated strains to tolerate drought. In terms of the ability to grow at high temperatures, all isolates were able to grow at 35°C and 40°C, and more than 80% of them also grew at 45°C, but only seven isolates could tolerate up to 50°C (Table 2). The temperature of 50°C is common in the summer in the sampling region, and this capability in the isolates shows their compatibility with this extreme situation. Moreover, all isolates grew in 1% and 3% salinity concentrations. More than 80% of the isolates could tolerate a salinity of 7%, but less than 50% of them sustained a salinity of 10%.

**Table 2:** Physiological characteristics of selected isolates obtained from oil-contaminated soils in Dehloran

**Isolates**	**Concentration of NaCl (%)**				**Temperature (°C)**				**Tolerance to drought potentials (MPa)**					
	**1**	**3**	**7**	**10**	**35**	**40**	**45**	**50**	**-0.05**	**-0.15**	**-0.3**	**-0.49**	**-0.73**
**NC39**	++	++	++	++	++	++	++	++	+	+	+	+	+	
**NC392**	++	++	+	-	++	++	-	-	+	+	+	+	+	
**NC393**	++	++	-	-	++	++	++	-	+	+	+	+	+	
**NC394**	++	++	++	+	++	++	++	-	+	+	+	+	+	
**NC395**	++	++	++	+	++	++	++	+	+	+	+	+	+	
**NB83**	++	++	++	+	++	++	+	++	+	+	+	+	-	
**NB832**	++	++	-	-	++	++	-	-	+	+	+	+	+	
**NB833**	++	++	++	+	++	++	++	++	+	+	+	+	-	
**NB834**	++	++	++	-	++	++	++	+	+	+	+	+	+	
**NB835**	++	++	-	-	++	++	++	-	+	+	+	+	-	
**NB84**	++	++	++	+	++	++	+	+	+	+	+	+	+	
**NB842**	++	++	++	+	++	++	++	-	+	+	+	+	+	
**NB843**	++	++	+	-	++	++	+	-	+	+	+	+	+	
**NB391**	++	++	+	-	++	++	++	-	+	+	+	+	+	
**NB392**	++	++	++	-	++	++	++	+	+	+	+	+	+	

(-) = no growth, (+) = moderate growth, and (++) = optimal growth.

**Figure 1:**
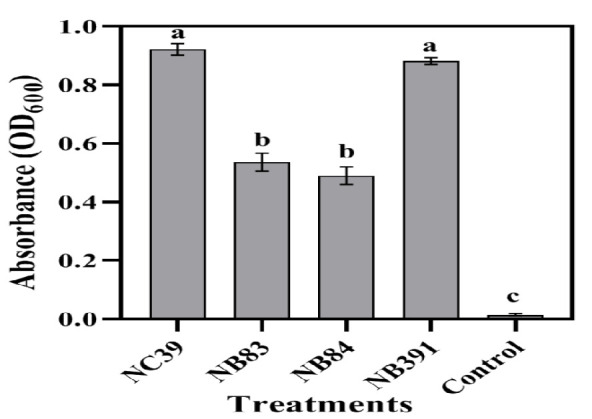
Optical density of the 7th-day grown cultures of the isolates when hydrocarbons of diesel were used as the sole carbon source. Bars on the columns indicate the standard error of the mean. Results were obtained from three repetitions of the experiments.

The efficiency of the four selected isolates in the degradation of diesel (5 g/L) as the sole carbon source from the aqueous medium was investigated. After 7 days of incubation, the growth of bacteria was checked by measuring the turbidity of the culture against its initial value at 600 nm. The increase of turbidity in cultures indicated the ability of bacteria to utilize diesel hydrocarbons as the carbon source. Among the 15 isolates used in the physiological characteristics experiment, four isolates of NC39, NB83, NB84, and NB391 were selected due to the suitable growth in the solid oil medium as well as growth in unfavorable environmental conditions. According to the results of Figure 1, the isolates of NC39 and NB391 showed the highest growth, and two isolates of NB83 and NB84 showed middle growth in the presence of diesel. 

**Figure 2:**
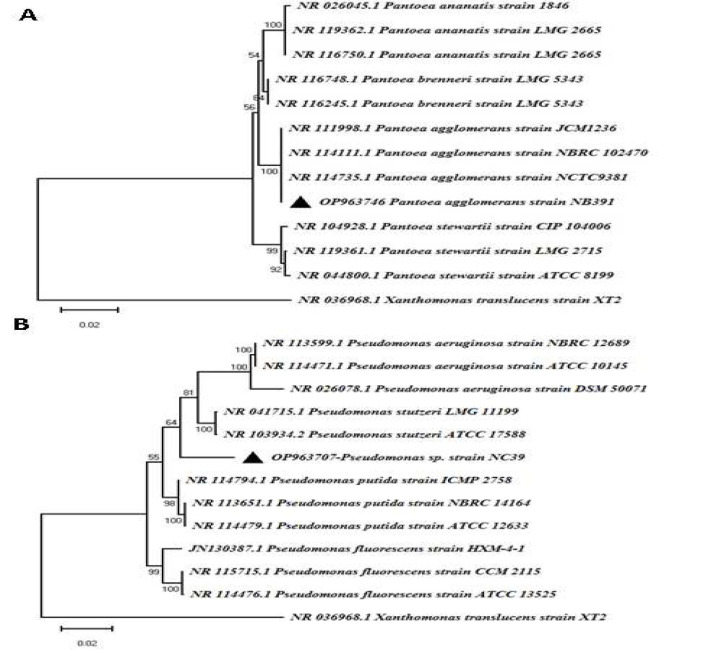
Phylogenetic dendrogram based on the 16SrRNA region of the selected isolates and some reference sequences obtained from GenBank by Neighbor-Joining method (1000 bootstrap replicates) in MEGA X software. A) NB391 isolate B) NC39 isolate. *Xanthomonas* *translucens* species is considered an outgroup.

Four selected isolates of NC39, NB83, NB84, and NB391, which passed the diesel degradation process, were used for phenotypic and biochemical identification (Table 3). Among the selected isolates, the best isolates (NC39 and NB391), in terms of their high ability to degrade diesel, were selected for molecular identification by the 16SrRNA region. After amplifying this region, bands of approximately 1500 bp appeared on the 1% agarose gel. The results of similarity using BLAST showed that isolate NC39 had 98% similarity to the *Pseudomonas* genus, but it was not able to distinguish between the species of this genus, so it was classified as *Pseudomonas* sp. It was deposited in GenBank with accession number OP963707. Additionally, NB391-isolate showed 99.7% similarity with *Pantoea* *agglomerans* species. It was deposited in GenBank with an accession number OP963746. In the phylogenetic analysis of the two studied isolates, they showed a high similarity with the reference sequences of their similar species obtained from the GenBank database (Fig. 2).

**Table 3:** Phenotypic and biochemical characteristics of selected isolates isolated from oil-contaminated soils

**Isolate**	**Gram reaction**	**Catalase**	**Oxidase**	**Urease**	**O/F**	**Shape**
**NC39**	**-**	**+**	**-**	**+**	**O**	**Bacilli**
**NB83**	**+**	**+**	**-**	**+**	**F**	**Coccus**
**NB84**	**+**	**+**	**+**	**+**	**O**	**Coccus**
**NB391**	**v**	**+**	**-**	**-**	**F**	**Bacilli**

## DISCUSSION

Exploiting the indigenous microorganisms is a promising approach to the bioremediation of petroleum pollution due to the compatibility of these microbes with the prevailing environmental conditions. Most studies have shown that bioremediation using indigenous bacteria is more reliable and easier to implement [8]. The current research sought to isolate and screen bacterial isolates indigenous to the highly-contaminated soils in mud pits and identify efficient isolates in degrading petroleum hydrocarbons. Moreover, the ability of isolates was screened to tolerate drought, salinity, and temperature stress. During drilling, mud with various ingredients, especially in particle size, crude oil, and salts, will be added to the mud pit. In addition to crude oil pollution, drilling mud ingredients strongly change the physical and chemical characteristics of the soil (Table 1). Our samples were sandy, which reduces their water-holding capacity. Besides this, the high concentration of long-chain aromatic and aliphatic hydrocarbons causes soil hydrophobicity. Therefore, moisture can be a limiting factor in the microbial activity of these soils. Salinity and high EC were also limiting factors for biodegradation in these soils [8, 23]. Microbes that have adapted to these extreme conditions have better ecological competence to re-establish in the same environment. 

The genus *Pseudomonas* is among the microorganisms whose degrading ability has been proven due to their ability to use various carbon sources, tolerance, and survival in exposure to hydrocarbon pollutants [24]. Various studies have identified *Pseudomonas* strains as bacteria that degrade diesel. For example, in a study by Niazy et al. (2016), after 10 days of culture at 30°C, results showed that *P. aeruginosa* I2 had the highest growth rate and diesel-oil degradation (88%) among all isolates [25]. Hong et al. (2005) isolated *P. aeruginosa* IU5 from oil-contaminated soil and identified it as a diesel-degrading bacterium. This strain degraded up to 60% of the diesel in 13 days [26]. Moreover, Shukor et al. (2009) characterized a *Pseudomonas* diesel-degrading strain that can grow optimally in 3.5% (v/v) diesel [27]. In this study, the isolate NC39, which was identified as *Pseudomonas* sp., showed a high growth rate in the medium containing 5 g/L diesel after 7 days. In addition, this isolate showed a high ability to tolerate high salinity (10%), temperature (50°C), and drought (-0.73 MPa) stress. Furthermore, the isolate NB391, which was considered to be *Pantoea* *agglomerans* in terms of biochemical characteristics and sequencing of the 16SrRNA, showed a high ability to consume diesel hydrocarbons. The genus *Pantoea* is well known as a member of microbial communities used in the bioremediation and biodegradation of hydrocarbons [28]. Research has proven the high potential of isolates from *P.* *agglomerans* to degrade petroleum hydrocarbons in soil [29-31]. The *P. agglomerans* NB391 was a facultative anaerobic bacterium (Table 3) that can also be active in anaerobic conditions usually occurring by closing the soil pores due to hydrophobic hydrocarbons. On the contrary, *Pseudomonas* sp. NC39 was obligate aerobic but had high urease activity, which can help the bacterium exploit external nitrogen sources, especially urea. A high C/N ratio is the limiting factor for microbial activity in soils contaminated with petroleum hydrocarbons [32, 33]. These different characteristics of the selected strains suggest their simultaneous application, which will be investigated in future research.

Environmental factors such as salinity, drought, and high temperature are the limiting factors in the microbial activity and biodegradation of petroleum hydrocarbons [13, 23, 34-36]. Considering the high salinity of some collected soil samples and the high temperature of the sampling region, it was necessary to evaluate the stability of the screened isolates in extreme environmental conditions such as salinity, drought, and temperature. According to the results, most isolates could tolerate high salinity levels, as high as 7%. The isolates of the present study could tolerate high temperatures (more than 45C), which could be due to their compatibility with the high temperature of the region. Regarding tolerance to drought potentials, the selected isolates tolerated a high potential (-0.73 MPa) of drought in the medium. Considering the effect of environmental drought on the biodegradation ability of bacteria [11], this potential indicates the high compatibility of these isolates. 

It seems that indigenous microorganisms acquired the ecological fitness to tolerate the unfavorable climate conditions of the region and the microclimate of the contaminated soils. Overall, the results of this research, while confirming the unfavorable conditions of the region and contaminated soils, showed that the obtained strains have proper capabilities to tolerate these extreme conditions and biodegrade petroleum hydrocarbons. Therefore, using these strains individually or in a consortium will be a promising approach to the biodegradation of pollution in these extreme environmental conditions that prevail in most oil-rich regions of the Middle East.
